# Long-time qingyan formula extract treatment exerts estrogenic activities on reproductive tissues without side effects in ovariectomized rats and via active ER to ERE-independent gene regulation

**DOI:** 10.18632/aging.102035

**Published:** 2019-06-19

**Authors:** Hong-Xia Zheng, Yuan Zhao, Ying Xu, Zi-Jia Zhang, Jing-jing Zhu, Yuan-Fang Fan, Na Lin

**Affiliations:** 1 Institute of Chinese Materia Medica, China Academy of Chinese Medical Sciences, Beijing, China; 2 Institute of Chinese Materia Medica, Shanghai University of Traditional Chinese Medicine, Shanghai, China

**Keywords:** reproductive target tissue, estrogenic effect, estrogen receptors, estrogen receptor antagonist ICI182,780

## Abstract

The reproductive tissues are negatively influenced by estrogens in hormone therapy. Qingyan formula ethanol extract (QYFE)’s estrogenic effects and safety on reproductive tissues after long-term administration and its mechanism via estrogen receptor (ER) pathway haven’t been studied. Here, we characterized its estrogenic effects using ovariectomized rats together with *in vitro* studies for further molecular characterization. Ovariectomized rats were treated with QYFE at doses of 0.7, 1.4, and 2.8g/kg for 12 weeks. The results showed QYFE has a potent estrogenic activity, as indicated by restoring the disappeared estrous cycle, antagonizing the atrophy of uterus, vagina and mammary gland, and the estrogen decline in circulation caused by ovariectomy. In addition, QYFE upregulated ERα and ERβ expressions and had a less stimulatory effect on PCNA and ki-67 antigen in reproductive tissues compared with estradiol valerate. QYFE components can bind to ERα and ERβ, significantly increased ERα/β-ERE luciferase reporter gene expression, upregulated the expressions of ERs, PR and pS2 in MCF-7 cells at protein and gene level. All these activities were significantly inhibited by the ER antagonist ICI182,780. QYFE’s estrogenic activity maybe mediated by stimulating biosynthesis of estrogen and increasing the quantity of ERs in target tissue and via active ER to ERE-independent gene regulation.

## INTRODUCTION

Perimenopausal syndrome including urogenital atrophy, decrease in sexual activity, psychological depression, osteoporosis, and vasomotor alterations, Which are caused by a drastic decrease in estrogen production in women. The hormonal replacement therapy (HRT) usually haven been taken to ameliorate most of these symptoms [1]. However, some side-effects such as vaginal bleeding, or an increased risk of cancer [2–5] were induced by HRT, many women refuse or discontinue HRT treatment. The uterus is a target of E_2_, without the addition of progestin E_2_ stimulates endometrial proliferation; which bring out endometrial hyperplasia and possibly lead to neoplasia [6]. In the vagina, epithelium is undergo proliferation and cornification by E_2_, lactobacillus use epithelium cells to produce lactic acid to keep the vaginal milieu acidic and then prevent ascending infections, which are the desired estrogenic effects [7]. Another undesired effect is stimulation of hyperproliferation of mammary gland tissue for E_2_ [2]. Therefore, perimenopausal women choose to seek alternative therapies from some functional plant extracts. Phytoestrogens are similar with mammalian estrogens on structurally and functionally, but with less side effects compared with synthetic HRT [8, 9]. Phytoestrogens have been considered as selective estrogen receptor modulators (SERMs), it could bind to estrogen receptors (ERs) and appear to have various estrogenic and antiestrogenic effects [10, 11].

Traditional Chinese medicines (TCM) have been used for treatment of peri-menopausal syndrome for hundreds of years in China and regarded as phytoestrogens resource. Post-menopausal women has been explained as a kidney deficiency in the TCM theory and can be treated by kidney-invigorating agents [12, 13]. QingYan formula (QYF) is recorded in the ShengJiZongLu during Song Dynasty in China and includes *Halitium, pricklyashpeel, Morinda officinalis, achyranthes bidentata and Cistanche deserticola.* QYF has been used to prevent and treat various related diseases of Kidney deficiency, and strengthen tendon and bone through nourishing kidney. In our previous study, QYF ethanol extract (QYFE) induced the growth and development of the uterus and vagina in immature mice within 7 day treatment [14]. Immature and ovariectomized (OVX) rats/mice model were recommends used for estrogenic activity test by the Organization for Economic Co-operation and Development [15]. Currently, the biological effects of QYFE on OVX rat after long-term oral administration is not characterized and whether QYFE causes few side effects, as reported phytoestrogens, or whether they have endocrine disruptors that endanger the uterus, vagina and mammary gland. Furthermore, the molecular mechanism of action of QYFE has not been revealed. In this study, we describe the estrogenic effects of QYFE using an in *vivo* model of OVX rats within 12-week treatment together with *in vitro* studies focusing on the classical ER-ERE signal transcriptional pathway for further molecular characterization. Molecular modeling, binding experiments, and ER α/β transcriptional activity and functional assays were performed to evaluate its further mechanism.

## RESULTS

### QYFE restored the estrus cycle of OVX rat

**Figure 1:**
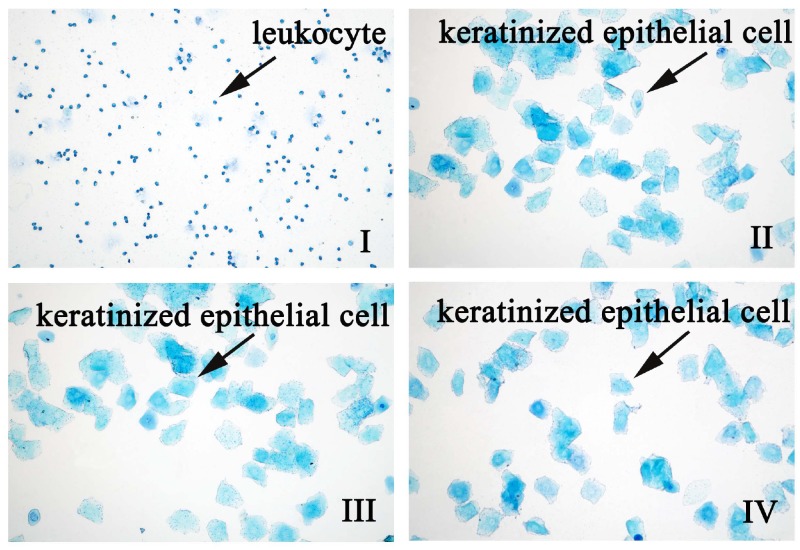
**The effect of QYFE on the estrous cycle in ovariectomized (OVX) rats.** EV refers to estradiol valerate, QYFE to Qing Yan Formula 70% ethanol extract. Representative photomicrographs taken at 200-X magnification. (**I**) OVX rats untreated; (**II**) Shan; (**III**) OVX rats treated with estradiol valerate (EV); (**IV**) OVX rats treated with 2.8g/kg QYFE.

To reveal the estrogenic activity of QYFE, a synthetic estrogen, estradiol valerate (EV) tablet worked as positive control, which is from DEL PHARM Lille S.A.S. The estrous cycle of all rats was monitored by inspection of vaginal epithelial cell smears. The vagina is covered by Malpighian pluristratified epithelium which marked sensitivity to estrogens. A high surge of estrogen level results in the full cornification of vaginal epithelial cells. As shown in Figure 1I, the estrous cycle of all the OVX rats without treatment was blocked at the diestrus stage and the smear exhibited only leukocytes, confirming the complete disappearance of ovaries and absence of endogenous estrogens. The oral administration of EV (Figure 1 III) or QYFE at three doses (Figure 1 IV) in OVX rats activated the cornification of epithelial cells after 10 days treatment, which indicate that the status of estrus in the OVX rats was restored. The sham-operated rats had a five-day normal estrous cycle (Figure 1 II).

### QYFE increased index of uterus and adrenal gland, and up-regulated serum E_2_

**Figure 2:**
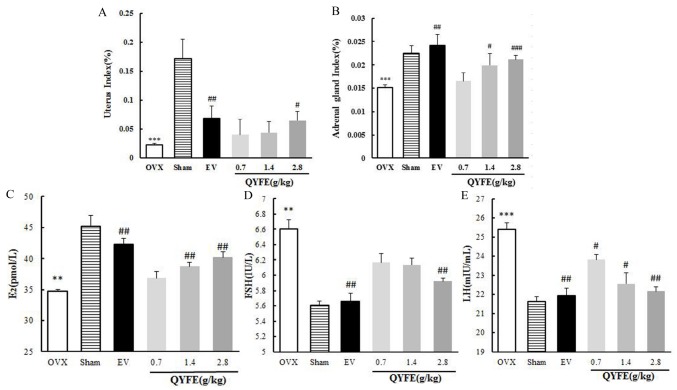
**The effects of QYFE on index of uterine and adrenal gland, and sex hormone in circulation.** The uterus index (**A**), adrenal gland index (**B**), serum levels of estradiol (E_2_) (**C**), luteinzing hormone (LH) (**D**) and follicle-stimulating hormone (FSH) (**E**) for OVX rats were measured at the end of the 12-week treatment period. Data are the mean and standard deviation (SD) of samples from 10 rats. P values are for the one-way analysis of variance (ANOVA). ^***^p < 0.001, compared with the sham group; #p < 0.05, ##p < 0.01, ###p < 0.001, compared with the OVX group.

QYFE treatment induced modest stimulatory effects on uterus weight in OVX rats (Figure 2A). QYFE had a trend of increasing weight with an increasing dose, and the highest dose, 2.8 g/kg, resulted in a significant difference with a 2.8-fold increase in weight compared with untreated OVX rats (p < 0.05). Compared to untreated OVX rats group, EV treatment induced a 3.0-fold increase in uterus index (p < 0.01).

Moreover, the mean adrenal gland index of OVX animals was significantly lower than that of sham group as shown in Figure 2B (p < 0.001). Compared with OVX rats, EV treatment dramatically increased the adrenal gland index (p < 0.01). There was a trend of increasing weight with an increasing dose of QYFE, the highest dose, 2.8 g/kg, caused a 0.4-fold increase in weight compared with untreated OVX rats (p < 0.001).

Ovariectomy is expected to result in significant inhibition of serum E_2_ and higher levels of LH and FSH compared to sham group rats. Treatment of OVX rats with EV, raised levels of circulating E_2_ by 22%, QYFE at dose of 1.4 and 2.8 g/kg increase 11%, and 16% than of that of untreated OVX rats respectively (all p<0.01) (Figure 2C). QYFE treatment induced a modest decrease in serum FSH level, and there was a significant difference only at the highest dose of QYFE and was comparable to the decrease induced by EV treatment (Figure 2D). Serum LH was significantly decreased in OVX rats treated with QYFE at any doses tried, or after treatment with EV. The down-regulating effects increased as the dose of QYFE increased. The LH concentration of OVX rats treated with QEFE at 2.8 g/kg or with EV, was around 14% lower than of that of untreated OVX rats, respectively (both p<0.01) (Figure 2E).

### QYFE antagonized the histological atrophy of uterus, vagina and mammary gland

**Figure 3:**
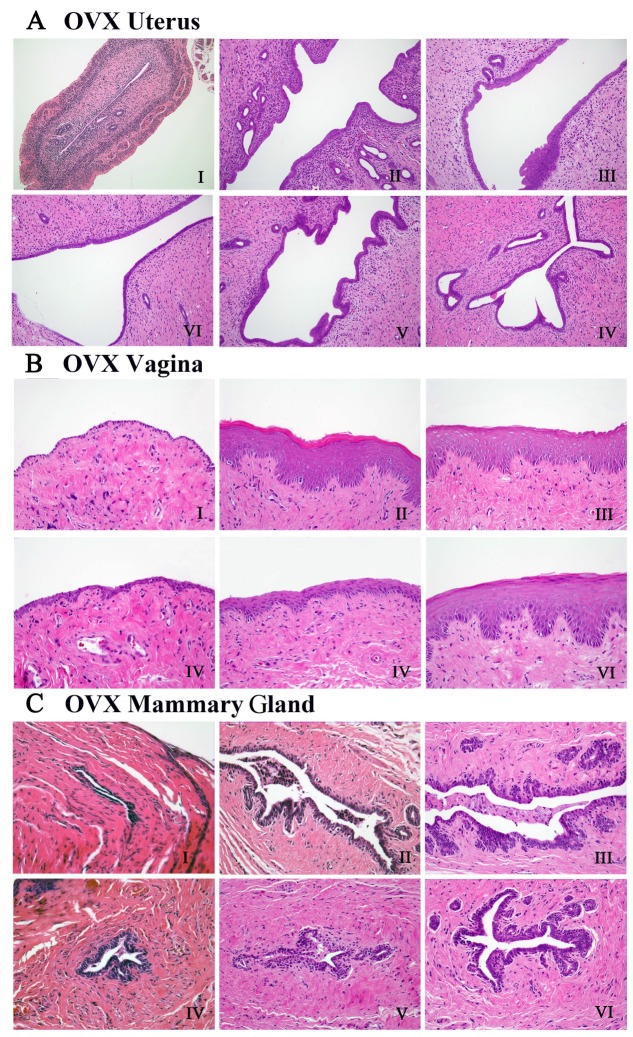
**The effects of QYFE treatment on the histology of uterus, vagina and mammary gland in the OVX rats.** Representative photomicrographs taken at 200-X magnification of uterine, 400-X magnification of vaginal and mammary gland sections in ovariectomized (OVX) rats. (**A**–**C**) are the histology of the uterus, vagina and mammary gland, respectively in OVX rats. The treatment groups in OVX rats are shown: (I) Untreated ovariectomized (OVX) rat; (II) sham-operated rat; (III) OVX rat treated with estradiol valerate (EV); and OVX rat treated with (IV) 0.7 g/kg, (V) 1.4 g/kg, and (VI) 2.8 g/kg QYFE.

Microscopic preparations of representative uteri from one animal per group was shown in Figure 3. Compared with sham controls, histological analysis of uterine sections in the untreated OVX rats revealed significant atrophy, the obvious degeneration of the cavities, endometrium and secretory glands (Figure 3AI). EV or QYFE treatment substantially restored uterine morphology of OVX rats, as indicated by the thickening of the uterine endometrium, the increased number of glands and more extended glandular cavities compared with untreated OVX rats (Figure 3AIII–VII), the uteri morphological findings of all animals were quantified and are presented in Table 1. In untreated OVX rats, the endometrium was composed of single layered columnar epithelial cells, and no mitotic activity was detected in epithelial cells. Endometrial cells were stimulated but no pathological signs in the QYFE medium group animals (Figure 3A IV–VI), such as hypertrophic and hyperplastic glands were detected. EV (Figure 3A III) induced estrogenic features, inducing the endometrial epithelium to become multilayered and hypertrophic, mitotic activity was present in the endometrial cells in most animals at various degrees. Thus, QYFE had a similar ability as EV to reverse the atrophy caused by ovariectomy.

Figure 3B shows microscopic preparations of representative vagina from one animal per group, the morphologic findings in vaginas of all animals were quantified and are presented in Table 1. In untreated OVX rat, only three to five cell layers were present, and these were composed of flattened cells with no cornification was observed in 10 of 10 rats. The EV-treated animals (Figure 3B III) displayed a typical squamous multilayered epithelium compared to untreated OVX rats. Approximately 10~15 cell layers with cornification were present in all 10 samples. In QYFE-treated animals, epithelium thickness and the cell layers were increased in some areas, and cornification was observed in 8 of 10 rats. An increased epithelial thickness and the number of cell layers (10 layers) were present in 2.8 g/kg QEFE treatment group (Figure 3B VI), and cornification was found in 9 of 10 animals.

**Table 1:** Quantitative data of histological feature in uterus and vagina of ovariectomized rats.

**Group**	**Uterus endometrial thickness(μM)**	**Uterusendometrial glands numbers**	**Vaginaepithelium cell layers**	**Vaginal thelium thickness (μM)**
OVX	387.25±150.54^***^	8.75±1.70^**^	1.50±0.571^***^	14.75±8.84^**^
Sham EV QYF0.7g/kg	2370.00±505.17 1279.00±490.77^##^ 1158.75±634.91^#^	50.00±23.16 13.50±7.18^#^ 15.50±3.41^#^	9.75±1.70 7.50±2.64^###^ 3.75±0.95^##^	162.5±70.41 91.50±49.33^#^ 28.25±9.94^#^
QYF1.4g/kg	1265.25±634.81^#^	15.25±5.59^#^	5.00±2.78^#^	48.75±24.10^#^
QYF2.8g/kg	1388.75±643.75^#^	21.25±9.87^#^	8.00±2.30^##^	88.25±58.6^#^

(^***^) *P* <0.001, (^**^) *P* < 0.01 and (^*^) *P* < 0.05, compared with the Sham group; (^###^) *P* < 0.001, (^##^) *P* < 0.01 and (^#^) *P* < 0.05, compared with OVX group.

As presented in Figure 3C, the mammary glands are composed of connective tissue, acini, and ducts, with the epithelial cells of the acini and ducts manifesting cubic or low columns in the sham operated rats. In OVX group, all epithelial structures appeared atrophic and there was scarce clusters of densely packed terminal structures in the deep of fat pad, many of which did not show lumina formation (Figure 3C I). EV or QYFE treatment induced abundant terminal epithelial structures, significantly increased mammary gland epithelium thickness, and resulted in formation of small lumina (Figure 3C III–VI).

Taken together, these results demonstrated QYFE has estrogenic activity and prompted further studies to elucidate the molecular mechanism, in particular to the effects of QYFE are mediated through ERs activation**.**

### Effect of QYFE on ERα, ERβ, PCNA and Ki67 receptors in uterus, vagina and mammary gland

**Figure 4:**
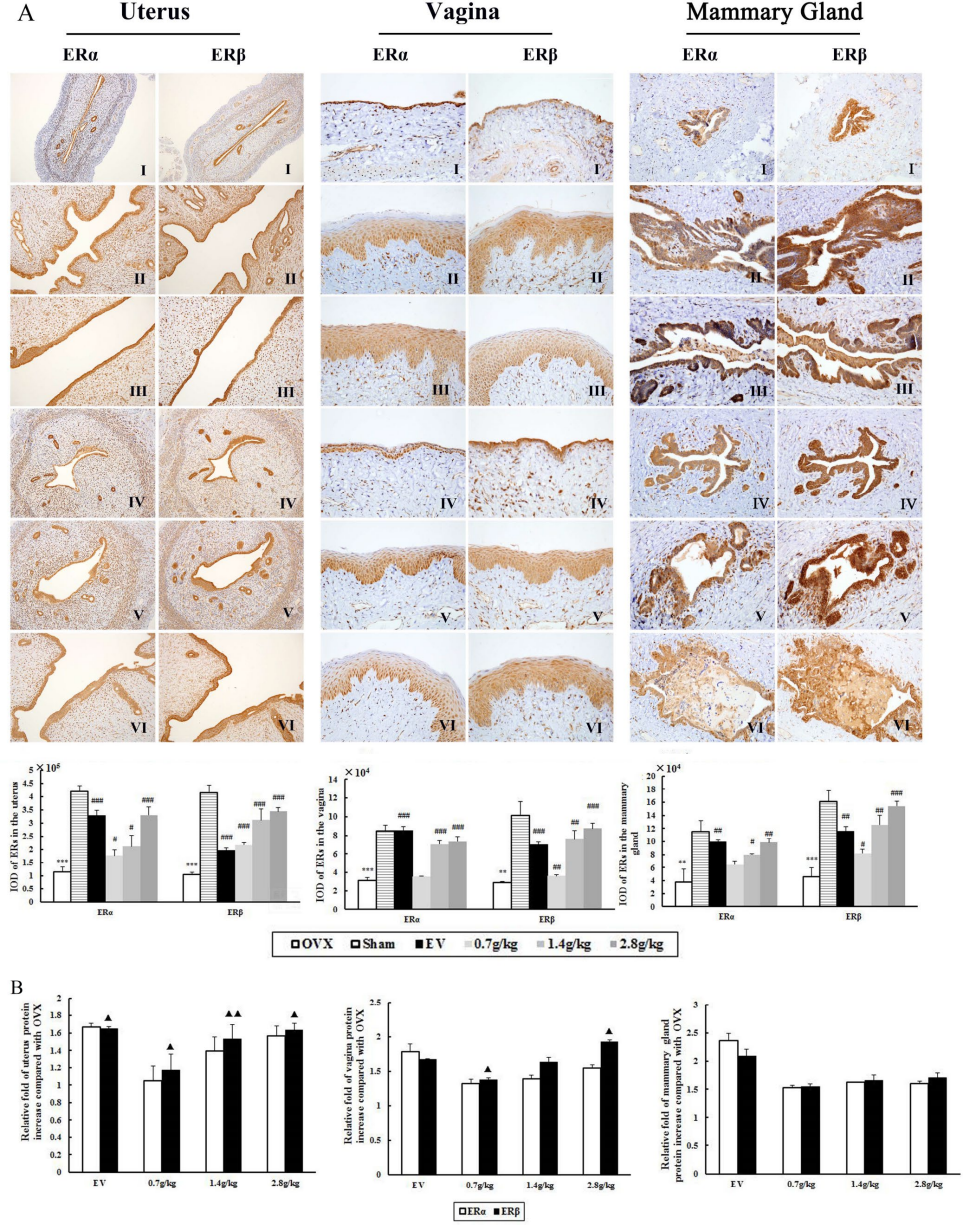
**The effects of QYFE treatment on the expression of estrogen receptor (ER) α and estrogen receptor (ER) β in the uterus, vagina and mammary gland.** (**A**) ERα and ERβ expression was assessed by quantitative immunohistochemistry. Representative photomicrographs taken at 200-X magnification of uterus and 400-X magnification of vagina and mammary gland sections from each treatment group are shown: (I) Untreated ovariectomized (OVX) rat; (II) sham-operated rat; (III) OVX rat treated with estradiol valerate (EV); and OVX rat treated with (IV) 0.7 g/kg, (V) 1.4 g/kg, and (VI) 2.8 g/kg QYFE. Data are the mean standard deviation (SD) of samples from 10 rats. ^***^ p< 0.001, compared with the sham-operated group; ###p < 0.001, ##p < 0.01, and #p < 0.05, compared with the OVX group. (**B**) Comparative Statistical analysis of ERα and ERβ upregulation to OVX group (▲) p< 0.05, compared with ERα protein relative increase of QEFE to untreated OVX.

The effects of QYFE treatment on ERs expression in the target tissue of OVX rats was assessed by immunohistochemistry. Representative sections and quantitative analysis from each treatment group are shown in Figure 4. The uterus, vagina and mammary gland of untreated OVX rats expressed minimal amounts of ERs (Figure 4AI), and treatment with either QYFE or EV caused a clear upregulation of ERα and ERβ (Figure 4AIII–VI), the high dose 28 g/kg QYFE induced the maximum increases (both p< 0.001). Besides, ERβ up-regulation was stronger than that of ERα in all target tissue of QYFE treatment (p<0.05 or 0.01), while EV treatment induced ERα up-regulation was stronger than that of ERβ in uterus and vagina (p<0.05 or 0.01) (Figure 4B). ERs were expressed in the uterus epithelial cells of the endomentrium, interstitial cells and smooth muscle cells in the QYFE-treated, EV-treated, or sham-operated groups, ERs were expressed in vaginal epithelial cells, squamous cells, and smooth muscle cells in the vagina. Epithelial cells in mammary gland duct positively expressed ERs. These data further support the indication that QYF mediates its activity *in vivo* through ERs.

**Figure 5:**
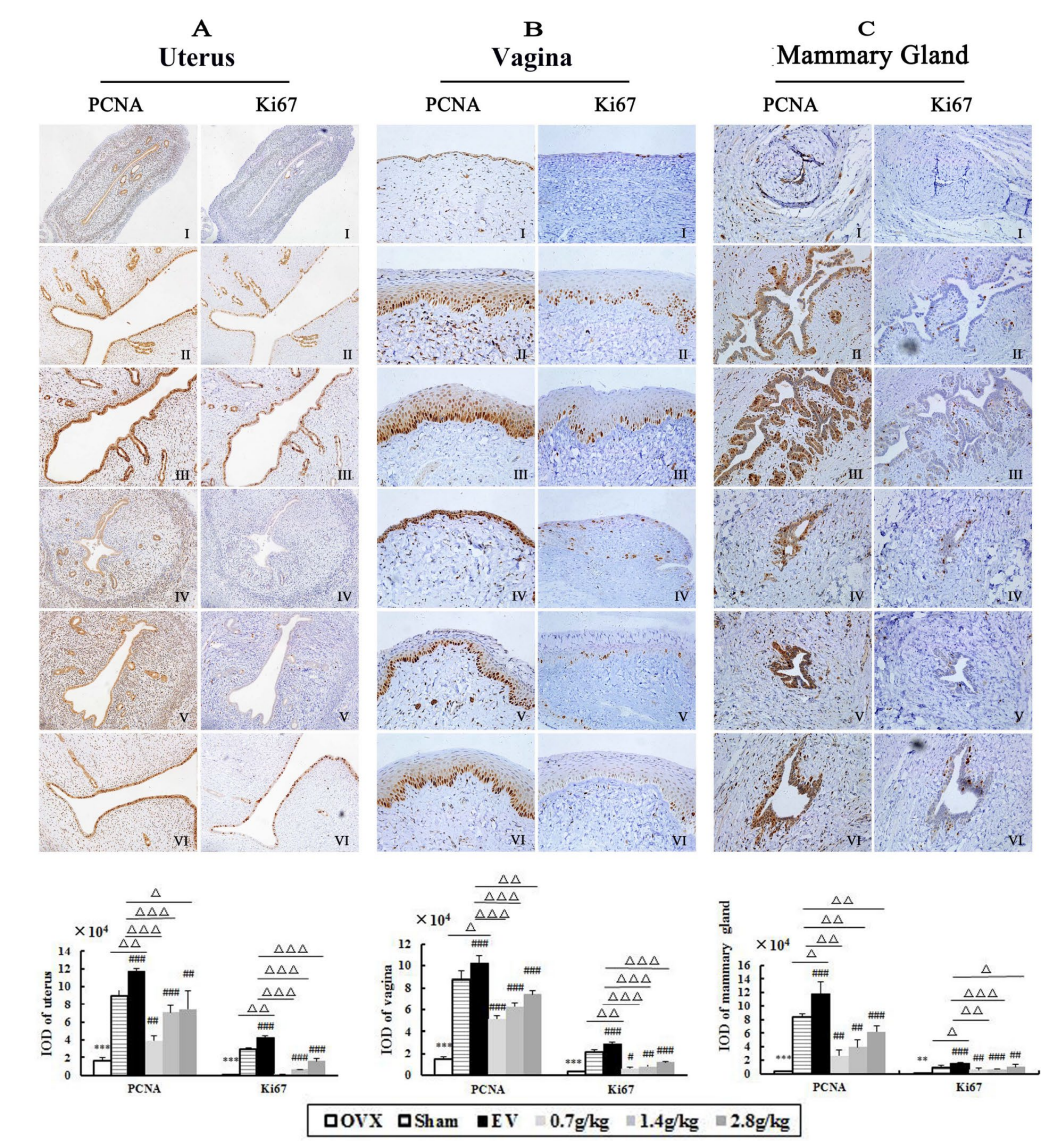
**The effects of QYFE on expression of PCNA or Ki67 in the uterus, vagina and mammary gland in OVX rats.** PCNA and Ki67 expression was assessed by quantitative immunohistochemistry. Representative photomicrographs taken at 200-X magnification of uterus (**A**) and 400-X magnification of vagina (**B**) and mammary gland (**C**) sections from each treatment group are shown: (I) Untreated ovariectomized (OVX) rat; (II) sham-operated rat; (III) OVX rat treated with estradiol valerate (EV); and OVX rat treated with (IV) 0.7 g/kg, (V) 1.4 g/kg, and (VI) 2.8 g/kg QYFE. Data are the mean standard deviation (SD) of samples from 10 rats. ^**^P<0.01, ^***^P<0.001, compared with sham; #p<0.05, ##p<0.01, ###p<0.001, compared with OVX group;△p<0.05, △△p<0.01, △△△p<0.001, compared with EV group

Figure 5 shown EV or QYFE treatment significantly stimulated PCNA and Ki-67 expression in uterus, vagina and mammary gland compared with untreated OVX rats (p< 0.01 or 0.001). It is worth mention that EV induced a remarkable upregulation of PCNA and Ki-67 compared with sham group (p<0.05 or 0.01), while QYFE resulted significant downregulation level in these parameters compared with sham group or EV treatment. (p<0.05, 0.1 or 0.01).

### QYFE increase ER subtypes expression in uterus, vagina, and mammary gland

**Figure 6:**
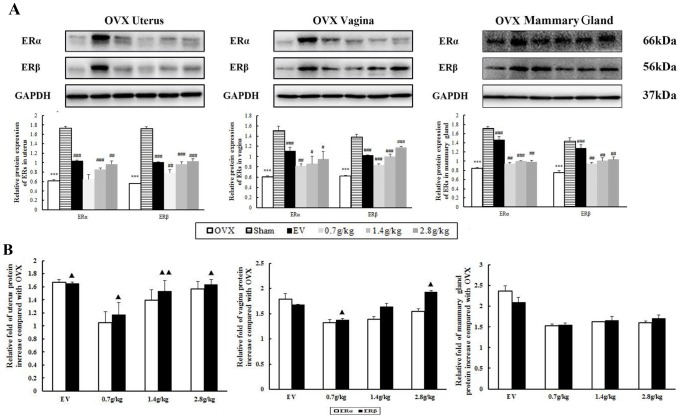
**The effects of QYFE on the expression of estrogen receptor (ER) α and estrogen receptor (ER) β at protein levels in uterus, vagina, and mammary gland of rats.** (**A**) Western blot analysis was carried out as described in Materials and Methods. Representative blots are shown above, and quantitative analysis is shown below. Values given are the mean standard deviation (SD) of three independent experiments. ^***^ p < 0.001, and ^**^ p < 0.01, compared with sham-operated; ###p<0.001, ##p< 0.01 and #p< 0.05, compared with the ovariectomized (OVX) group; (▲) p< 0.05, compared with ERα protein relative increase to Control. (**B**) Comparative Statistical analysis of ERα and ERβ upregulation to OVX group. (▲) p< 0.05, compared with ERα protein relative increase of QEFE to untreated OVX.

Further evidence for the interaction of the QYFE with ERs was performed by determining the effects on ERs expression in target tissues by western blotting. As shown in Figure 6, compared with the sham group, both protein expressions of ER α and ERβ were significantly decreased in the uterus, vagina, and mammary gland in OVX rats (all p<0.001). EV or QYFE treatment induced significant up-regulation of ERα and ERβ expressions in target tissues, with a dose-dependent manner in QYFE treatment. The 2.8 g/kg QYFE induced the largest up-regulation of protein expression with a 0.6-fold increase in ER α (p<0.01) and a 0.8-fold increase in ERβ (p <0.001) in the uterus, compared with untreated OVX rats. Meanwhile, the 2.8 g/kg QYFE treatment also up-regulated protein expression of ER α 0.6-fold (p<0.01) and ERβ 0.9-fold (p<0.01) in the vagina and increased protein expression of ERα 0.2-fold (p<0.05) and ERβ 0.4-fold (p<0.01) in the mammary gland, respectively. Meanwhile, Figure 6B Shown ERβ up-regulation was stronger than that of ER α in uterus and vagina of QYFE treatment (p<0.05 or 0.01), while EV treatment induced ERα up-regulation was slight stronger than that of ERβ.

### *In vitro* studies

#### QYFE stimulated MCF-7 cell proliferation and no influence on MDA-MB-231 cell

**Figure 7:**
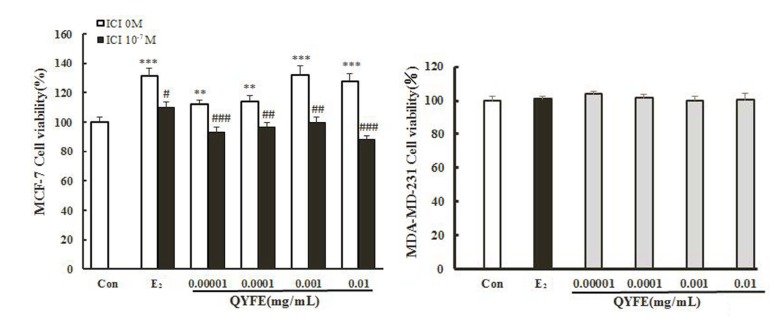
**Effect of QYFE on viability of MCF-7 cells and MDA-MB-231 cell.** Cell proliferation was carried out as described in the Materials and Methods. Results are expressed relative to the growth of cells treated with 1% dimethylsulfoxide (DMSO). Data are the mean ± standard deviation of quadruplicate analyses, expressed relative to that of treatment with 0.1% DMSO. ^***^p < 0.001, ^*^p < 0.05 compared to Con; ###p < 0.001, compared to QYFE or 0.01 μM 17β-estradiol (E_2_).

To investigate the molecular basis of QYFE activity in more detail, ER positive MCF-7 human breast cancer cells were used as a model because they are dependent on estrogen for growth. As shown in Figure 7, Intermediate concentrations of QYFE and 0.01μM 17β-estradiol both induced proliferation with a max 24.8% and 30% increase, respectively compared to DMSOcontrol, indicating the estrogenic activity of the QYFE extracts, which were significantly inhibited by the specific ER antagonist ICI 182, 780. In contrast, QYFE has no significant effect on the growth of ER negative MDA-MB-231 cells from dose of 0.00001 to 0.01 mg/mL. Our results showed that QYF caused a significant enhancement in cell proliferation of ER positive breast cancer cells, while had no significant effect on ER negative breast cancer cells.

#### QYFE stimulated the binding effect of ERα and ERβ

**Figure 8:**
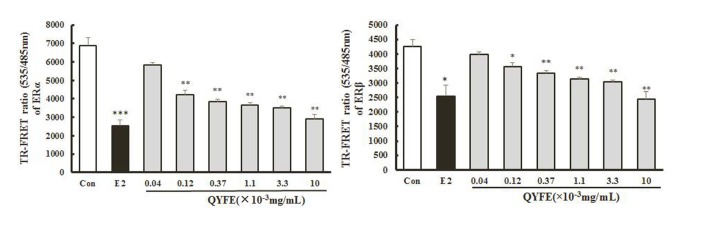
**Effect of QYFE on ability of ERα and ERβ binding.** Each data point represents the mean±standard of triplicate samples.^*^p < 0.05,^**^p < 0.01,^***^p < 0.001 compared to Con.

Specific ER antagonist ICI182, 780 inhibited the estrogenic activities of QYFE on the MCF-7 cell proliferation. We next examined whether QYFE could directly bind to ER using a TR-FRETER competitive assay. The polar fluorescence polarization value indicates the fluorescence expression of nuclear receptor which is not drug-substituted, and the smaller the fluorescence polarization value, the higher the ability of the drug to bind to the ER. Figure 8 showed QYFE could bind to human ERα and ERβ ligand binding domain (LBD) in the dose range of 0.12 ~ 10 μg/mL. As the concentration increased, the combination was enhanced.

#### QYFE induced both ERα and ERβ transcriptional activity

HEK 293 cells with stably transfected with the hERα/β-ERE-luciferase plasmid were used to measure the formation of functional hERα/ β -ERE complexes in response to treatment with the QYFE. Results are expressed relative to expression in DMSO-treated cells. QYFE induced both ERα and ER β -ERE luciferase activity increase, QYFE at 0.001mg/mL resulted in a 2.0-fold increase in ERα and a 1.4-fold increase in ER β luciferase activity, which were comparable to the 1.4-fold increase in ERα and 1.9-fold increase in ER β luciferase activity induced by 17 β -estradiol at 0.01μM. These effects were inhibited by the specific ER antagonist ICI182, 780, resulting in 82% and 63% inhibition of ERα and ER β expression in cells treated with 0.001mg/mL QYFE, respectively, which is obviously decreased comparable to that observed with 17 β-estradiol treatment. These data indicate that QYFE clearly has estrogenic activity that is mediated through the ERs-ERE activation.

#### QYFE increased the protein and gene expression of ERα, ERβ, PR and pS2 in MCF-7 cells

**Figure 9:**
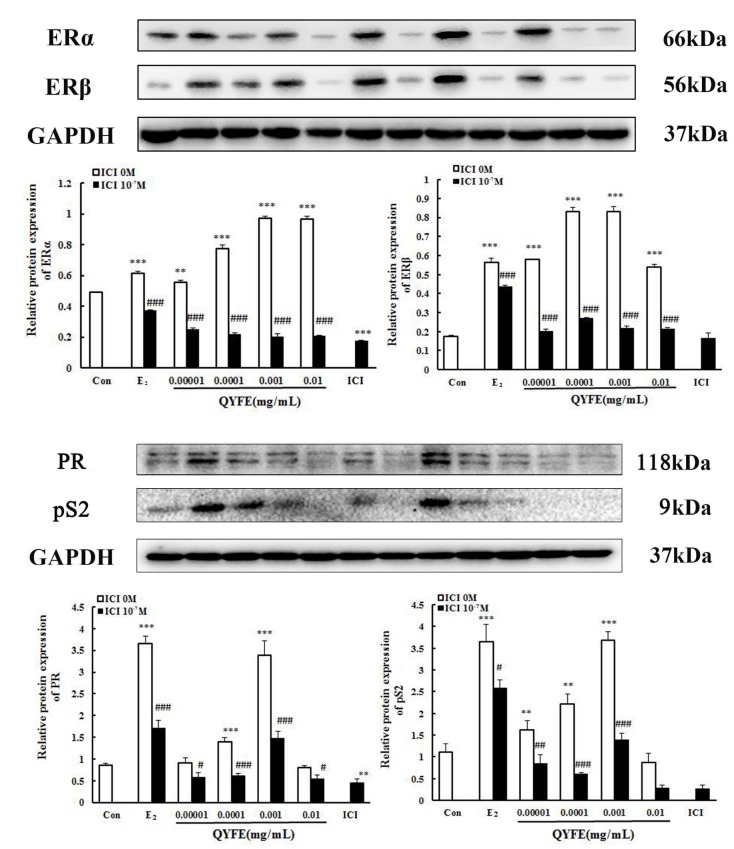
**Effect of QYFE on the protein levels of estrogen receptors (ER) α and ERβ, PR and ps2 in MCF-7 cell.** Western blotting analysis was carried out as described in the Methods ^*^P<0.05, ^**^P<0.01, ^***^P<0.001, compared with con; ^#^*P*<0.05, ^##^*P*<0.01, ^###^*P*<0.001, compared with 17β-estradiol (E_2_/) QYFE.

Further evidence for the interaction of the QYFE with the ER system was sought by determining the effect on ER subtype, the estrogen related PR and pS2 expression in MCF-7 cells by western blotting and RT-PCR. As Figure 9 shown, the western blotting results indicate that 0.01mg/mL QYFE upregulated ERα, ERβ, PR and pS2 expression by 0.98-fold, 3.7-fold, 2.9-fold and 2.3-fold, respectively, respectively. The expressions were upregulated were up-regulated slightly more by QYFE at the concentration of 0.001 mg/mL than by 17 β-estradiol. The effects of QYFE were significantly inhibited by the specific ER antagonist ICI182, 780 with a 80% decrease in ER α, a 74% decrease in ER β, a 56% decrease in PR and a 62% decrease in pS2. Our results indicating that the activities of QYFE, compared to 17 β-estradiol, is mediated via the ER pathway. Moreover, ER β upregulation by QYFE was stronger than that of ER α (p< 0.05)

**Figure 10:**
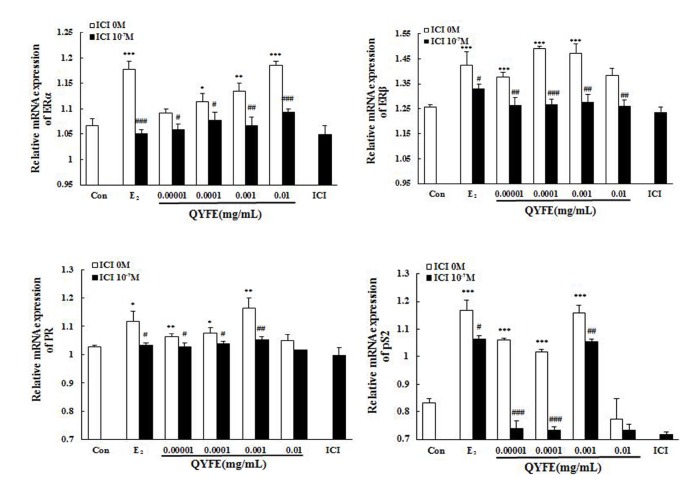
**Effect of QYFE on the the gene expressions of estrogen receptor (ER) α, ER β, PR and pS2 in MCF-7 cells.** Real-time PCR analysis was carried out as described in the Methods.^*^P<0.05,^**^P<0.01, ^***^P<0.001compared to Con; ^#^*P*<0.05, ^##^*P*<0.01, ^###^*P*<0.001 compared to 17β-estradiol (E_2_) or QYFE.

The change of ER subtype, pS2 and PR mRNA production in theMCF-7 cells treated with QYFE based on GAPDH. The results as shown in Figure 10, treatment with either 17β-estradiol or QYFE (0.00001~0.01mg/ml) induced significant up-regulation of ERα, ERβ, PR and pS2 on mRNA levels. The higher dose of QYFE, 0.01 mg/ml, resulted in the largest up- regulation of these gene expression compared with the control (p < 0.001 or p < 0.01). Besides, in QYFE+ ICI group, the mRNA expressions of ERα ERβ, PR and pS2 decreased at the concentration of 0.00001~0.01mg/ml (p < 0.001, p < 0.01 or p < 0.05), with a similar effect to E_2_ + ICI group

## DISCUSSION

This study aimed to investigate the estrogenic effects and safety of QYFE on the uterus, vagina and mammary gland in OVX rats after 12 week long-term administration, and molecular mechanism of its effect via the estrogen receptor pathway. The results showed that QYFE restored the disappeared estrous cycle, antagonized the atrophy of uterus, vagina and mammary gland, and estrogen decline in circulation caused by ovariectomy. In addition, QYFE upregulated ERα and ERβ expressions in reproductive target tissues, and had a stimulatory effect on PCNA and ki-67 antigen in reproductive tissues. QYFE’s estrogenic activity maybe mediated by stimulating biosynthesis of estrogen and increasing the quantity of ERs in target tissue and via the ER-ERE-independent signal transduction pathway.

Previous studies have revealed the effects of QYFE on estrogen target tissues in immature mice after a short time 7-day application. Reports about effects after long-term administration are scarce, particularly concerning safety. This study, we observed PCNA and Ki-67 expression in the uterus, vagina and mammary gland of OVX rats treated with QYFE. PCNA is a nuclear protein, which is expressed in proliferating cells during the S phase of the cell cycle, as a useful tool in mammary, cervical, and endometrial cancer prognosis research [16, 17]. Protein of proliferation intensity (Ki-67-antigen) is an excellent marker for determining the growth cell fraction of a given cell population [18]. In the EV-treated group, the number of PCNA- and Ki-67-positive cells in the uterus, vagina and mammary gland significantly increased compared to the untreated and Sham group. QYFE has a clear stimulatory effect on PCNA and ki-67 analysis in the target tissues compared to the control untreated group, but lower than that of sham group (Figure 5). The results suggest that QYFE treatment in a long time is maybe safer in the reproductive target tissues than EV treatment. Moreover, ERα is required for cervical and vaginal cancers development, ERα -selective agonists could increase the risk of carcinogenesis for a long-term use [19]. There are reports that ERα and ERβ produce opposite effects on human breast cancer cell proliferation and tumor formation [20]. ERα mediates the breast cancer-promoting effects of estrogens, and ERβ mediates its inhibitory effects. The ratio changes of ERα : ERβ during the process of tumorigenesis with ERα increase and ERβ decrease, which has been observed in relation to breast [21], colon [22], and prostate [23] cancers. We found that QYFE upregulated ERβ expression significantly more than ERα in the reproductive target tissue, especially in the uterus and vagina (Figure 4) These results suggesting that QYFE could induce agonistic or antagonistic effects depending on target organs, such as SERM, and QYFE was safe for reproductive target tissue, maybe owing to balance the ratio of ERα :ERβ.

Estrogens are synthesized in the ovary or testis and adrenal gland [24]. The adrenal gland becomes the principal tissue for secreting estrogen after ovariectomy. The increased weight of adrenal gland and serum estrogen concentration in QYFE higher doses suggests that the effect of QYFE may be mediated through the hypothalamus-pituitary-adrenal axis and stimulate biosynthesis of estrogen in the adrenal gland. FSH and LH levels as markers of the estrogenic effect of QYFE on the hypothalamic-pituitary axis where the gonadotropin-releasing hormone (GnRH) pulse generator resides in the hypothalamus [25]. Under the absence of estrogens, the GnRH pulse generator is over-active, which leads to high serum FSH and LH release from the in pituitary of post-menopausal woman [26]. QYFE at higher doses promoted E_2_ release and diminished the ascending FSH and LH levels in OVX rats, suggesting either the long loop effect of QYFE directly on the hypothalamus or the short-loop effect on the pituitary gland.

**Figure 11:**
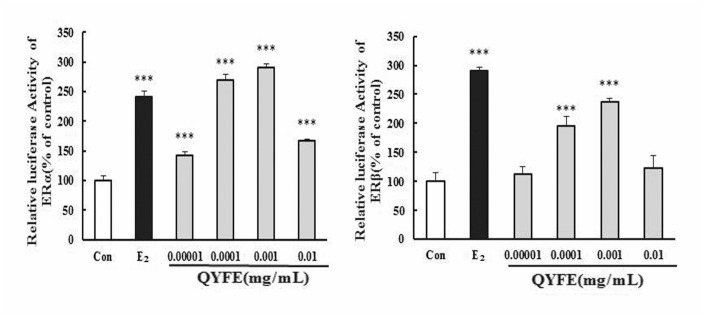
**Effects of QYFE on the activation of estrogen receptors (ER) α and ERβ in HEK293T cells.** The effect of QYFE on estrogen receptors α and β activity in the transiently transfected HEK293T-ERα and HEK293T-ERβ cells was investigated by measuring reporter gene-coupled luciferase activity. ^***^P<0.001 compared to Con; ^#^*P*<0.05, ^##^*P*<0.01, ^###^*P*<0.001 compared to 17β- estradiol or QYFE.

Estrogen mediates its actions by binding to ERs and inducing a major conformational change, resulting in the estrogen-ER complex to relocate to the nucleus to bind to its cognate DNA response element (ERE) located in the promoter/enhancer regions of target genes and allowing the regulation of gene transcription [27, 28]. Molecular modeling and TR-FRET ER competitive assay demonstrate that QYFE components are the ligands of both ERα and ERβ (Figure 8). QYFE significantly increased ERα or ERβ-ERE luciferase reporter gene expression (Figure 11), and up-regulated the expressions of ERs, PR and pS2 in MCF-7 cells at protein and gene level (Figures 9, 10). The MCF-7 cell line expresses ERs and is dependent on estrogen for proliferation in monolayer culture [29, 30]. Our results showed that QYFE only significantly induced the viability of MCF-7 cells and no influence on the ER-negative MDA-MB-231 cells, QYFE is more sensitive to ER-positive MCF-7 human breast cancer cells (Figure 7), which indicates the induced of MCF-7 cell proliferation by QYFE maybe mediated by ER. Progesterone receptor (PR) and restrogen-regulated pS2 gene are directly regulated by E_2_ through ER and serve as a reliable marker for estrogen responsive activity [31–33]. Additionally, the positive agonist effects by QYFE were significantly inhibited by the ER antagonist ICI182,780. These results demonstrated that QYFE exerts ER agonist activity through competitively bind to ER, inducing ER upregulation and active ER to ERE-independent gene regulation.

In summary, our data demonstrate that QYFE antagonize the atrophy of uterus, vagina and mammary gland and without side effects in ovariectomized rats. QYFE’s estrogenic activity maybe mediated by stimulating biosynthesis of estrogen and increasing the quantity of ERs in target tissue and via the ER-ERE-independent signal transduction pathway. Further studies are in progress to investigate the use of QYFE as an effective dietary supplement for the improvement of quality of life in peri-menopausal women.

## MATERIALS AND METHODS

### Chemicals and reagents

17β-Estradiol (E_2_), ICI182,780 and 3-(4,5-dimethylthiazol-2-yl)-2,5-diphenyltetrazoliumbromide (MTT) were obtained from Sigma (St. Louis, MO, USA);Dulbecco modified eagle medium (DMEM)and other cell culture supplements were purchased from GIBCO (Grand Island, NY, USA); Dimethyl sulfoxide (DMSO) was from Sinopharm Chemical Reagent Co., Ltd.(Beijing, China). Charcoal-dextranfetal bovine serum (CD-FBS) was bought from biological industries (Kibbutz Beit-Haemek, Israel); Estrogen receptor α antibody, estrogen receptor β antibody, Ki-67 antibody and pS2 antibody were purchased from Abcam (Cambridge, UK). LanthaScreen TR-FRET ERα and ERβ competitive binding assay kits were obtained from Life Technologies (Carlsbad, California, USA); The dual-luciferasereporter assay system were provided from Promega (Madison, USA). All other chemicals solvents used for high-performance liquid chromatography (HPLC) were of HPLC grade.

### Preparation of ethanol extract of QingYanFormula (QYF)

QEF was prepared as described in ancient medical books. Briefly, the Five herbal medicines Halitium, Pricklyash Peel, Morinda officinalis, radix achyranthis bidentatae and herba cistanches (1:1.5:2:2:2) were extracted with ethanol/water (70/30) for twice, 5 h each time. The solvent ratio was 1:10 w/v for the first time and 1:8 w/v for the second time. The extracts were drying to powder to constant weight under reduced pressure and the yield of extraction was 48.9% (w/w). The representative estrogenic activity chemical compositions (Supplementary Figure 1) of monotropein (0.0189%), asperuloside (0.0109%), acteoside (0.0160%) and β-ecdysone (0.2374%) in QYF ethanol extract (QYFE) were determined by HPLC analysis. The combined QYFE were concentrated *in vacuo* and dissolved in DMSO (1 g/mL). All of the samples were stored at -4°C.

### In vivo studies

#### Animal treatment

All experimental procedures and animal management were approved and performed by China Academy of Traditional Chinese Medicine.

Sixty female Sprague-Dawley rats (200±10g) were obtained from Experimental Animal Center of Academy of Military Medical Sciences (Certifiate No. SCXK [Jun] 2012-0004). All rats were underwent either ovariectomy or sham-operation (Sham) under general anesthesia with 0.3% (m/V) pentobarbital sodium (1mL/100g rat, i.p.). OVX rats were removed ovaries bilaterally, while the rats of sham-operated group were removed fat near the ovary. All ovariectomized rats were checked by daily vaginal epithelium cell smear analysis, in which 5 consecutive days of leukocytes were indicative of constant dioestrus and successful ovariectomy. The OVX rats were assigned into six groups randomly, Ovariectomized without treatment (OVX, n=10), sham operated (sham, n=10), ovariectomized rats treated with 0.1 mg/kg estradiol valerate (EV, n=10), and OVX rats treated with QYFE intragastrically at a daily dose of 0.7 g/kg, 1.4g/kg, or 2.8 g/kg for 12 weeks. Dose calculations followed guidelines correlating dose equivalents between humans and laboratory animals, on the basis of ratios of body surface area [34]. Untreated control ovariectomized and sham rats received distilled water only. All animals were maintained under controlled conditions of (24±2°C) and humidity (55±5%), a 12-hour light/dark cycle, and allowed free access to food and water.

### Analysis of vaginal cornification, serum and target tissues index

The vaginal smear of each rat was taken daily every day during 12 weeks treatment. The vaginal lavage was fixed with 95% ethanol for 10 min and stained with methylene blue for 10 min. Vaginal epidermal cells were observed by microscopy, and keratinized vaginal cells were taken as being indicative of estrus [35, 36].

Animals were sacrificed after 12 weeks of treatment. Blood was collected from the abdominal aorta and serum for analysis of E_2_ and luteinizing hormone (LH) by enzyme linked immunosorbent assay (ELISA). (Beijing Xinfangcheng Biotechnology, China) [37]. The sensitivities of the three ELISA assays were 1.0 pg/mL, 1.0 mIU/mL and 1.0 ng/mL respectively and not soluble structural analogues with other cross-reaction, and all the intra-assay and inter-assay variation of each hormonal assay were less than 9% and 15%.

The uterus and adrenal gland were removed and weighed. The left horns of the uterus and the upper portion of the vagina were stored at −80°Cfor analysis by western blot, and the right horns of the uterus and the rest of vagina were fixed with 4% polyoxymethylene for 24 h for staining with hematoxylin and eosin (H&E) and immunohistochemistry.

### Histology

Tissue sections 4 μm-thick of uterus, vagina and mammary were cut mounted on polylysine coated slides and stained with Hematoxylin & Eosin (H&E) for microscopy [38]. The histological structure changes were observed, including the thickness of the uterine endometrial and number of uterine endometrial glands, vaginal epithelial cell layer and vaginal thelium thickness.

### Immunohistochemistry

The immunohistochemistry protocol and semi-quantitative analysis were carried out as described in our previous study [39, 40]. Estrogen receptor- α (ERα), estrogen receptor-β (ER β), anti-proliferating cell nuclear antigen (PCNA) and ki-67 immunoreactivity were visualized by diaminobenzidine (DAB). The following antibodies were used: rabbit anti-ER α monoclonal antibody (1:200, ab32063, Abcam Biotechnology, UK), rabbit anti-ER β polyclonal antibody (1:400, ab3577, Abcam Biotechnology, UK), rabbit anti-PCNA polyclonal antibody (1:2000, ab18197, Abcam Biotechnology, UK), and rabbit anti-ki-67 monoclonal antibody (1:200, ab16667, Abcam Biotechnology, UK). The Image-Pro Plus 6.0 System image analysis system was used for quantitative analysis.

### Western blot assay

The protein of uterus, vagina and mammary gland were separated using RIPA lysis buffer (Beijing Pulilai Gene Technology Co., Ltd., cat no: B1007). The western blot protocol and semi-quantitative analysis were carried out as previous study described [39]. The antibody of rabbit anti-ERα monoclonal antibody (dilution 1/1000, ab32063, Abcam Biotechnology, UK), rabbit anti-ER β polyclonal antibody (dilution 1/2000, ab3577, Abcam Biotechnology, UK), rabbit anti-glyceraldehyde 3-phosphate dehydrogenase (GAPDH) monoclonal antibody (dilution 1/5000, ab181602, Abcam Biotechnology, UK) were used. The reactive bands were visualized by electrochemiluminescence (ECL; Pierce, Rockford, IL). All experiments were done in triplicate. Visualization of protein bands was quantified by Alpha Ease FC (Fluorchem FC_2_) software.

### *In vitro* studies

#### MTT assay of MCF-7 and MDA-MB-231

The MCF-7 and MDA-MB-231 cell lines were purchased from XieheCell Research Institute of Peking Union Medical College (from the American Type Culture Collection[ATCC]), cultured in Dulbecco’s modified eagle’s medium (DMEM)supplemented with10% fetal bovine serum (FBS), penicillin/streptomycin (100 U/mL and 100 μg/mL), and incubated at 37 °C under 5% CO_2_ incubator. Two days prior to treatment the cells were sub-cultured in phenol red-free DMEM containing 5% charcoal-stripped FBS to improve the sensitivity of MCF-7 cells to estrogen, and then MCF-7 cells and MDA-MB-231 cells were seeded at the density of 5 × 10^3^cells/180 μL/well in 96-well plates, respectively. Cells were preincubated overnight in estrogen-depleted medium and test samples of QYFE extract (20 μL, at 0.0001~0.1mg/mL in 1% DMSO, the final concentration of DMSO in the cell delivery system is 1‰), 17β-estradiol, QYFE with ICI182, 780 and 1‰ DMSO solvent blank, then incubated for 48 h. Cell proliferation was evaluated at 492 nm by performing a MTT assay, as described Percent growth induction was calculated as a percentage of the average response of the DMSO control samples. Results reported are the mean ± standard deviation of four replicate determinations from a representative assay at least three times [41].

### ERs competitive ligand-binding assay

To confirm binding affinity of QYFE and ER_S_, PolarScreen™ ERα and ERβ Competitor Assay kits were used (Catalog nos. A15882, A15890, Life technologies^TM^, Carlsbad, CA, USA). In brief, series dilutions of QYFE (10, 3.3, 1.1, 0.37, 0.12, 0.04, 0.013, 0.004 μg/mL) were competed with Fluormone^TM^ GS1 Green for binding with terbium labeled ERs-LBD on a 384-well plate. One hour later after incubation at room temperature, the fluorescence intensity was detected on a microplate reader (Excitation: 340 nm; Fluorescein emission: 535 nm; Terbium emission: 485 nm; Envision^TM^, PerkinElmer). The final data were shown by normalizing the signal of fluorescein to that of terbium [42].

### Transfection and reporter assay of estrogen receptor-subtype selectivity

HEK 293 cells were stably transfected with human estrogen receptor α/β (hER α/ β) and the estrogen response element (ERE) plasmid (kindly provided from Professor Yung-Chi Cheng, Yale University), the formation of functional ER α/β -ERE complexes was evaluated by the luciferase reporter assay system from Promega (WI, USA). The cells were maintained in phenol red-free DMEM with 5% dextran-coated charcoal-stripped fetal calf serum to eliminate any estrogenic source before treatment and then seeded (8 × 10^5^ cells/180 μL/well) in 96-well plates. The cells were exposed to different concentrations of QYFE (0.00001~0.01mg/mL) with or without ICI182,780, 17β-estradiol treatment served as positive control, all tests were in three replicate wells. After 24 h of incubation, cells were lysed, and the lysates were used in measurements of luciferase activity. Results reported are the mean standard deviation of four replicate determinations from a representative assay [40].

### Western blot assay

MCF-7 cells were cultured in DMEM with 5% charcoal-dextran stripped FBS for 1 day to eliminate any estrogenic source before treatment, then the cells were treated with QYFE (0.00001~0.01mg/mL), 17β-estradiol (0.01 μM) with or without 0.1 μM ICI182, 780, 0.1% DMSO treatment as negative control. After 48h, all cells were harvested protein. Rabbit anti-ER α monoclonal antibody (dilution 1/1000, ab32063, Abcam Biotechnology, UK), rabbit anti-ERβ polyclonal antibody (dilution 1/2000, ab3577, Abcam Biotechnology, UK), rabbit anti-pS2 monoclonal antibody (dilution 1/1000, ab92377, Abcam Biotechnology, UK), rabbit anti-PR monoclonal antibody (dilution 1/1000, ab133526, Abcam Biotechnology, UK) were used, and rabbit anti-glyceraldehyde 3-phosphate dehydrogenase (GAPDH) monoclonal antibody (dilution 1/5000, ab181602, Abcam Biotechnology, UK) was used as internalcontrol. Finally incubated with goat anti-Rabbit IgG polyclonal antibodyHRP-Labelled (1:4000; C1309; Beijing Pulilai Gene Technology Co., Ltd.). All determinations were carried out in triplicate. Visualization of protein bands was accomplished using Immobilon Western Chemiluminescent HRP Substrate (Millipore, Billerica, MA, USA) [39].

### Quantitative real-time polymerasechain reaction (PCR)

Total RNA of MCF-7 was extracted using Common plant total RNA extraction kit (Cat#A010400, Beijing Aipbai Biotechnology Co., Ltd., Beijing, China) according to the manufacturer’s manual. RNA concentration was measured with a UV spectrophotometer (Colibri, Titertek-Berthold, Bad Wildbad, Germany) at a wave length of 260 nm and280 nm [43]. The total RNA (2μg) was reverse transcribed to cDNA using the iScript™ cDNA Synthesis Kit (Cat#170-8891, Bio-Rad, Hercules, California, USA) following the manufacturer’s instructions. The specific transcripts were quantified by quantitative real-time PCR using Taq SYBR® Green qPCR Premix (Cat#EG15135, Yugong Biolabs, Lianyungang, Jiangsu, China) and analyzed with an CFX96™ Real-Time System (BIO-RAD, Hercules, California, USA). Gene-specific primers were designed by DNAMAN 7.0 and used for ERα (forward, 5'-CCT CCCTGAACTTGCAGTAA-3'; reverse,5'-CCTGCTCCTTTCAACTACCA-3')[44], ERβ (forward, 5'-AGTCCCTGGTGTGAAGCAAG-3; reverse, 5'-CATCCCTCTTTGAACCTGGA -3')[44], PR (forward, 5'-AGCCAGAGCCCACAATACAG-3'; reverse, 5'-CCCACAGGTAAG GAC ACCAT-3'), pS2 (forward, 5'-AGAAGCGTGTCTGAGGTGTC-3'; reverse, 5'-GCAAATAA GGGCTGCT GTT-3')[45], and β -actin (forward, 5'-GCACCACACCTTCTACAATGA-3'; reverse, 5'-GTCATCTTCTCGCGGTTGGC-3'). The mRNA levels of ERα, ER β, PR and pS2 were normalized to β -actin mRNA levels. PCR was performed as 94°C for 3 min, followed by 40 cycles of 94°C for 15 sec, and 60°C for 1min. The quantification data were analyzed with ABI Prism analysis software. The relative mRNA expression was calculated with the comparative cycle threshold (*C_t_*_)_ method.

### Statistical analysis

The SPSS software version 11.0 for Windows (SPSS Inc, Chicago, IL, USA) was used for statistical analysis. All data were expressed as mean ± standard deviation and were analyzed by one-way analysis of variance (ANOVA) followed by least significant difference (LSD) or Dunnett’s T_3_ test. Differences were considered statistically significant when p was less than 0.05.

## Supplementary Material

Supplementary Figure 1
