# Supplementary figures and images for: Long-time qingyan formula extract treatment exerts estrogenic activities on reproductive tissues without side effects in ovariectomized rats and via active ER to ERE-independent gene regulation
Source: Aging (Albany NY). 2019 Jun 19;11(12):4032–49. doi: 10.18632/aging.102035 (PMC6628985; doi:10.18632/aging.102035)

SUPPLEMENTARY FIGURE

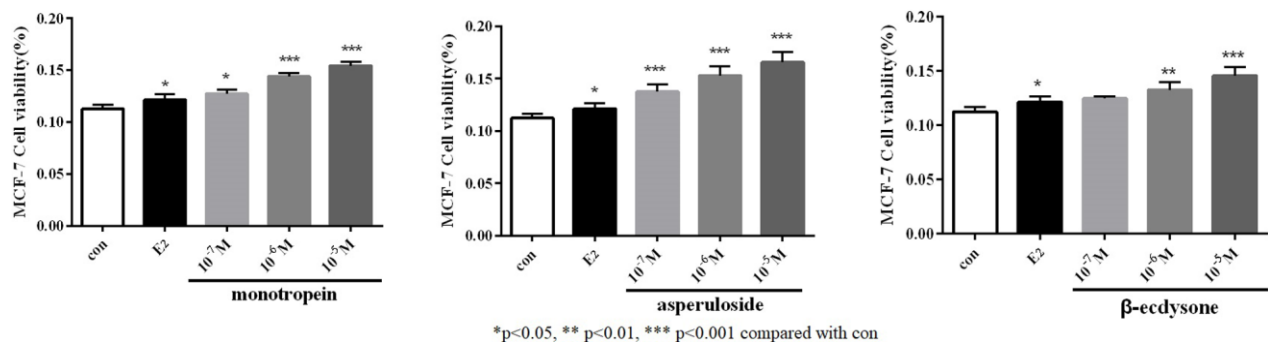

Supplementary Figure 1.

Supplement: Supplementary Figure 1 [file aging-11-102035-s001.pdf]
